# Patient-Perceived Benefit of Treatment in Polypoidal Choroidal Vasculopathy: A Pilot Study

**DOI:** 10.3390/ijerph17176378

**Published:** 2020-09-02

**Authors:** Rituparna Ghoshal, Sharanjeet Sharanjeet-Kaur, Norliza Mohamad Fadzil, Somnath Ghosh, Nor Fariza Ngah, Roslin Azni Abd Aziz

**Affiliations:** 1 Optometry and Vision Science Program, Faculty of Health Sciences, University Kebangsaan Malaysia, Kuala Lumpur 50300, Malaysia; rituparna.ghoshal@skf.edu.in (R.G.); norlizafadzil@ukm.edu.my (N.M.F.); 2 Department of Optometry, Supreme Institute of Management and Technology, Mankundu Hooghly, West Bengal 712123, India; 3 Department of Allied Health Sciences, Brainware University, Barasat, Kalkata, West Bengal 700125, India; somnath4ab@yahoo.co.in; 4 Department of Ophthalmology, Hospital Shah Alam, Persiaran Kayangan, Seksyen 7, Shah Alam 40000, Selangor, Malaysia; drfarizangah@gmail.com (N.F.N.); roslinazni@gmail.com (R.A.A.A.)

**Keywords:** polypoidal choroidal vasculopathy, patient-perceived treatment outcome, vision-targeted quality of life

## Abstract

Polypoidal choroidal vasculopathy (PCV), a subtype of neovascular age-related macular degeneration, requires repeated treatment. The objective of this pilot study was to evaluate and compare vision-targeted quality of life (QOL) at baseline and after 6 months of treatment in patients with PCV. Naive PCV patients were recruited. Visual functions assessed were distance visual acuity (DVA), near visual acuity (NVA), contrast sensitivity (CS), reading speed (RS), and QOL at baseline and after 6 months of treatment. Thirty patients (average age of 67.62 ± 8.05 years) revealed mean DVA and NVA improvements of 0.24 logMAR and 0.30 logMAR, respectively. Mean CS and RS improved by 0.39 log contrast and 25.58 words per minute, respectively. The National Eye Institute Visual Function Questionnaire 25 (NEI-VFQ-25) composite score significantly increased from a baseline of 66.73 ± 13.74 to 73.54 ± 14.26. Twenty-eight of the patients showed overall improvement in QOL score by 5 units or more or remained stable. Subscales of NEI-VFQ-25 significantly improved, with general vision, mental health, and role difficulties improving by 10 or more units. The present pilot study reports a significant improvement of QOL in PCV patients after 6 months of treatment, with mental health, role difficulties, social functioning, and distance vision activities being the most improved subscales.

## 1. Introduction

Today’s healthcare industry places a high focus on a patient-centered model. Thus, the measurement of patients’ perception of disease impact has been emphasized in healthcare for the last three decades. The impact of eye diseases on patients’ “real-life setting” is measured using several tools for assessing vision-targeted quality of life. The term “vision-targeted quality of life (QOL)” is governed by, but is not the same as, visual function. It represents the extent to which an individual’s visual status impacts his or her daily living activities along with social, emotional, and economic well-being. Thus, vision-targeted QOL is considered as one of the assessment tools used to measure the effect of eye disease and a patient’s treatment outcome [1,2].

Polypoidal choroidal vasculopathy (PCV) is an important and prevalent differential diagnosis upon retinal angiography of patients suspected of neovascular age-related macular degeneration (n-AMD) among Asian populations [3,4,5,6]. In a recent meta-analysis, Lorentzen et al. [7] reported the pooled prevalence of PCV to be 8.7% among white individuals with exudative AMD. Sharanjeet-Kaur et al. [8] reported that PCV can cause notable impairment of visual functions including distance and near visual acuity, contrast sensitivity, and reading speed. Thus, the disease restricts functional abilities of the individual. Furthermore, PCV requires a repeated treatment schedule causing frequent visits to the hospital, which may pose significant emotional and financial burden to the affected individuals as is reported in patients with n-AMD undergoing anti-VEGF treatment [9,10]. Previously, vision-targeted QOL was assessed in PCV-affected individuals [11,12]. However, the QOL of PCV patients after treatment has not yet been reported. Comparison between baseline and a 6-month outcome of vision-targeted QOL score in PCV patients will reveal the patients’ perceived benefits of the treatment process and subsequently will emphasize the need for long-term treatment expenditure in PCV. Therefore, the objective of the present study was to evaluate and compare vision-targeted QOL at baseline and after 6 months of treatment in patients with PCV. 

## 2. Materials and Methods 

### 2.1. Subject Recruitment

Patients clinically diagnosed with PCV by a senior retinal consultant (R.A.A.A.) were selected from the Ophthalmology Clinic of an AMD referral public hospital in Malaysia. The diagnosis of PCV was confirmed using the Japanese study criteria where findings of polyps and/or a branch vascular network on indocyanine green angiography (ICGA) is considered as a gold standard for PCV diagnosis [13]. However, in cases where ICGA was not performed due to reasons such as a patient’s allergy to indocyanine, optical coherence tomography (OCT) parameters such as sharp retinal pigment epithelium detachment (RPED) peak, double-layer sign, multiple retinal pigment epithelium detachment (RPED), RPED notch, hyporeflective lumen representing polyps, and hyper-reflective intraretinal hard exudates were taken into consideration. Among the 6 features of spectral domain OCT, the first two features and at least one of the other features sufficed for the diagnosis of PCV; in the absence of the first two features, the diagnosis of PCV was also made when at least 3 of the other features were present simultaneously [14]. Any retinal pathology other than PCV, such as other types of AMD, diabetic retinopathy, retinal vein occlusion, central serous retinopathy, or a macular hole; any history of treatment (antivascular endothelial growth factor injection, photodynamic therapy, or laser) for PCV; and cognitive, hearing, or motion impairment were excluded from the study.

### 2.2. Sample Size Determination

According to the developer of the National Eye Institute Visual Function Questionnaire 25 (NEI-VFQ-25), a sample size of 251 is required to find a 5-unit difference between pre- and postintervention NEI-VFQ-25 composite scores [15]. Thus, 10% of the sample size (251), that is, a minimum of 25 patients, was needed to conduct this pilot study.

### 2.3. Treatment of the Study Eyes

All patients included in this study received a first dose of intravitreal anti-vascular endothelial growth factor (anti-VEGF) (0.5 mg ranibizumab) (Lucentis; Genentech, South San Francisco, CA, USA) in the study eye at baseline. This is usually combined with a first session of photodynamic therapy (PDT) with verteporfin within the first week of anti-VEGF. Subsequently, the study eye received anti-VEGF injection at the second and third months of treatment. However, PDT was deferred in eyes with poor vision, larger lesions, multiple polyps, foveal fibrosis, and thinned fovea [16,17]. All the patients were followed up with monthly, and treatment strategy of each patient was reviewed by two senior retinal consultants (R.A.A.A. and N.F.N.).

### 2.4. Visual Functions of Study Eyes

All patients had undergone a comprehensive evaluation of visual functions, including distance and near acuity (DVA, NVA), contrast sensitivity (CS), and reading speed (RS), at baseline and 6 months after the first treatment. A 4-m Early Treatment Diabetic Retinopathy Study (ETDRS) logMAR chart was used to measure DVA. If any patient was unable to read the chart at 4 m, the chart was then moved to 3, 2, or 1 m until the patient could read the top line. Then, the visual acuity calculation was done according to the test distance used. Near visual acuity (NVA) was recorded as the smallest print size that was correctly read by the patient with best spectacle correction in logMAR using a UiTM Malay related-word reading chart at a standard distance of 40 cm. Visual acuity was recorded in logMAR for ease of analysis. Contrast sensitivity (CS) was measured using a Pelli–Robson chart at 1 m. Contrast score was based on the contrast of the last triplet in which two or more letters were correctly read by the patient and was recorded as such. Reading speed (RS) was measured using a UiTM Malay related-word reading chart by words per minute. It is a validated Malay language reading chart that has previously been described [18]. Reading speed was measured using the method recommended by the chart developers [18].

### 2.5. Assessment of Quality of Life

The National Eye Institute Visual Function Questionnaire 25 (NEI-VFQ-25) is a well-recognized tool for assessing vision-targeted quality of life of the visually impaired population. This widely used questionnaire that reveals individuals’ self-perceived visual function in different subscales [19] has been translated and validated in different languages [20,21,22,23]. Furthermore, having been proven as a reliable and valid tool to assess QOL in patients with AMD [24,25], it has been extensively employed for measuring vision-targeted quality of life of AMD populations including PCV [12,26,27,28,29,30]. It has been reported that NEI-VFQ-25 is a responsive and sensitive measure of vision-related function in neovascular AMD populations.

In the present study, the National Eye Institute Visual Function Questionnaire 25 (NEI-VFQ-25) in Bahasa Malaysia was used to assess quality of life (QOL). The NEI-VFQ-25 has three parts. The first part consists of 4 questions about general health and vision, the second part has 12 questions measuring difficulty with activities, and the third part assesses individuals’ response to vision problems with 9 questions. It measures vision-targeted quality of life with 12 subscales: general health (1 item), general vision (1 item), ocular pain (2 items), near-vision activities (3 items), distance-vision activities (3 items), social functioning (2 items), mental health (4 items), role limitations (2 items), dependency (3 items), driving (2 items), color vision (1 item), and peripheral vision (1 item). The NEI-VFQ-25 has been translated and validated in different languages. The Bahasa Malaysia version was developed in 2014 and has been validated to measure the vision-related quality of life among the Malay-speaking population [23]. The NEI-VFQ-25 in Bahasa Malaysia was administrated by a single interviewer at baseline and after 6 months of treatment. All questions were read with the corresponding answer options. Questions that were not clear for the patient were repeated when requested. Patients were given adequate time to understand and answer each of the questions. To avoid possible bias by the information obtained during the eye examination, patients completed the interview prior to seeing the optometrist or ophthalmologist.

### 2.6. NEI-VFQ-25 Scoring

The NEI-VFQ-25 was calculated using the standard scoring algorithm as proposed by the developers. Two steps were followed in this process. In the first step, original numeric values from the questionnaire were re-coded according to the scoring rule provided by the developers. Thus, item responses were transformed to a scale of 0–100. Here, a higher score represents a better quality of life. In the second step, items in each subscale were averaged together according to the table provided by the developers. Thus, a total of 12 subscales was obtained. 

To calculate the composite score of NEI-VFQ-25, a simple average of vision-targeted subscale scores, excluding the general health, were averaged. That is, a total of 25 vision-targeted questions were included while calculating the composite score. However, for a missing response (for example, when a question on driving was marked as missing) all the answered subscales’ scores were averaged.

Ethical approval was obtained from the Medical Research Ethics Committee of National Medical Research Register (NMRR-16-1965-31826 (IIR)) and Universiti Kebangsaan Malaysia Research and Ethics Committee (UKM 1.5.3.5/244/NN-186-2014), following the tenants of Helsinki. Written consent was obtained from all the participants. 

### 2.7. Statistical Analysis 

All the data were analyzed using SPSS software. Descriptive statistics and paired t-tests were employed to compare the visual functions and NEI-VFQ-25 composite scores between baseline and after 6 months.

## 3. Results

Although a minimum sample size of 25 patients was required for this pilot study, 30 PCV patients with newly diagnosed PCV were enrolled in this prospective longitudinal research from December 2016 to March 2017. They were evaluated for the patient-perceived benefit of treatment outcome after 6 months. 

Therefore, a total of 30 patients receiving treatment in an AMD referral public hospital in Malaysia were administered the NEI-VFQ-25 at baseline and 6 months after treatment. Twenty-four patients received combination therapy, while 6 patients received ranibizumab monotherapy. The mean age of the patients was 67.62 ± 8.05 years, and the ages ranged from 51 to 88 years. There were 17 male and 13 female patients. Among them, 12 were Malay, 14 were Chinese, and 4 were Indian. All the visual functions including DVA, NVA, CS, and RS improved significantly from baseline (*p* < 0.05) (Table 1).

**Figure 1:**
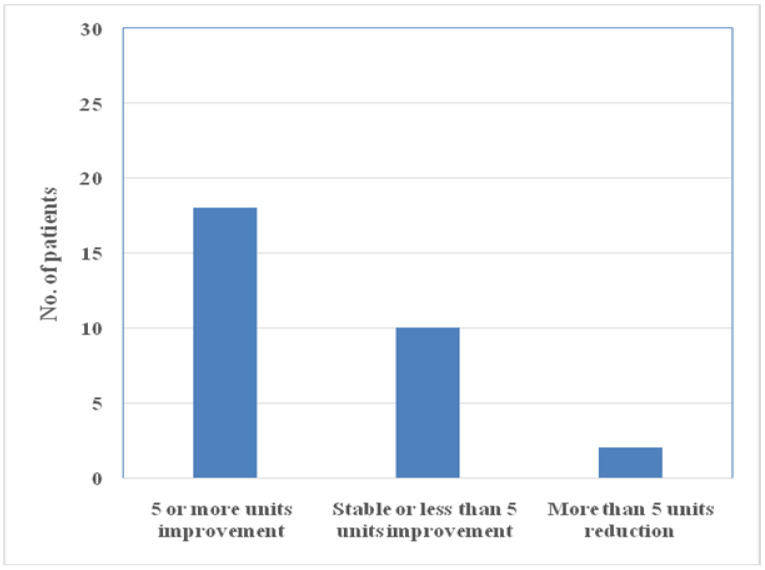
Change in National Eye Institute Visual Function Questionnaire 25 (NEI-VFQ-25) composite scores.

The overall QOL score significantly increased from the baseline of 66.73 ± 13.74 to 73.54 ± 14.26 (*p* < 0.001). A total of 28 (93.33%) of the patients showed overall improvement in the QOL score of 5 units or more or remained stable (Figure 1). Eighteen patients showed an improvement of 5 or more units, 10 patients showed an improvement of less than 5 units, and 2 patients showed a reduction of more than 5 units in NEI-VFQ-25 composite scores.

While assessing the correlation between the change of NEI-VFQ-25 composite score and change in visual functions after treatment, changes in DVA and NVA showed significant correlations (*r* = 0.55 and 0.42, respectively; *p* < 0.05) with change in NEI-VFQ-25 composite score. 

Different subscales of NEI-VFQ-25, including general vision, distance activities, dependency, mental health, role difficulties, and social functioning, were significantly improved (*p* < 0.05). Among them, mean general vision, mental health, and role difficulties improved by 10 or more units (Table 2).

## 4. Discussion

Literature on vision-targeted QOL of PCV is significantly limited. Fenwick et al. reported that the impact of PCV on QOL is similar to that of typical AMD [11]. Furthermore, Ghoshal et al. found no significant difference between mean NEI-VFQ-25 scores of typical n-AMD and PCV patients, although both groups exhibited low NEI-VFQ-25 composite scores [12]. However, the effect of treatment of PCV eyes on vision-targeted QOL is yet to be established. Thereby, this is the first study to evaluate the impact of treatment on the quality of life of PCV patients.

It has been proven that vision-targeted QOL shows maximum correlation with the better-seeing eye [31], and the majority of the patients of the present study had undergone treatment in the worse eye. However, the present study reported that the mean vision-targeted QOL improved significantly, with a mean difference of 6.81 points in the NEI-VFQ-25 composite score, after 6 months of treatment in patients with PCV seen in an AMD referral public hospital in Malaysia. This is similar to the results of Heier et al., who reported a NEI-VFQ-25 composite score improvement by 4.5 to 6.7 units in n-AMD eyes treated with a different regimen of ranibizumab and aflibercept after 1 year [32]. Likewise, several other studies reported improved vision-targeted QOL score in n-AMD patients after treatment [1,2]. Similarly, Chang et al. reported clinically meaningful changes in vision-related function scores even though the better-seeing eye was treated in less than half of the patients in many of the treatment groups of the study [1].

Furthermore, while Suner et al. (2009) used MARINA and ANCHOR data to report that NEI-VFQ-25 scores should change by 4 to 6 points to represent a clinically meaningful change in QOL in an AMD population [28], 60% of patients in the present research achieved at least 5-point improvements in NEI-VFQ-25 composite score after 6 months of treatment. Furthermore, the NEI-VFQ-25 composite scores of 30% of the patients remained stable, and only two patients showed reduced NEI-VFQ-25 composite scores after treatment. Both of these patients had poor vision (DVA of 0.9 logMAR or worse) and did not show any improvement in the majority of visual functions post-treatment. This could be a possible reason for reduced QOL score after 6 months. Likewise, different subscales, including general vision, distance activities, dependency, mental health, role difficulties, and social functioning, were significantly improved in the present study after 6 months. This is similar to the results of previous studies that reported statistical improvement in the different subscales after anti-VEGF therapy in patients with n-AMD [1,2].

However, other vision-specific activities did not improve significantly, including near activity, driving, color vision, and peripheral vision. This could be because of the fact that in the majority (90%) of the patients, the nontreated eye was the better eye in which there was no change after the treatment. Thus, activities like driving, reading, and finding objects on a shelf, which are more vision-demanding, might have remained the same. 

Conversely, it is important to note that the majority of the subscales that measure a person’s psychological, emotional, and social well-being improved significantly in the present study. Such subscales include mental health, role difficulties, dependency, and social functioning. In recent research, it has been shown that patients undergoing treatment for n-AMD are at risk for anxiety, mental stress, and depression [33]. Furthermore, McCloud et al. reported anxiety regarding injections, new limitations to lifestyle, and uncertainty about treatment procedure being some of the concerns of patients with n-AMD undergoing anti-VEGF injections in at least one eye [34]. This can affect the psychological well-being of the affected individual. Matamoros et al. reported that mental health and dependency were some of the most affected NEI-VFQ-25 subscales in patients with n-AMD undergoing anti-VEGF [29]. However, McCloud et al. further explained that the understanding of the treatment pattern by patients undergoing anti-VEGF treatment may help them to recover [34]. Armbreche et al. reported significant improvement in many quality of life aspects such as anxiety related to AMD and outdoor independence, even in the presence of progressive loss of vision [35]. Similarly, Chang et al. reported significant improvement of role difficulties, dependency, and mental health even when half of the study patients were treated for the worse eye [1]. Likewise, Bressler et al. reported that scores for mental health, dependency, and role difficulties improved from 6.2 to 15.7 points, with mental health being the most recovered subscale, after 1 year of treatment with anti-VEGF in n-AMD patients [2]. In agreement with the previous studies, the present results further indicate that even if the worse eye is affected by PCV, patients experience a significant impact on QOL; after treatment, there is significant improvement in vision-targeted QOL, including in subscales representing mental health, role difficulties, dependency, and social functioning. 

The major limitation of this study is a small sample size. An increased sample size will enhance our understanding of the effect of PCV treatment and enable the effects of various factors, including treatment type and regimen, to be assessed. Furthermore, the present study reports a significantly positive impact of treatment on PCV eyes after 6 months. However, following up with these patients for 1 to 2 years will help in assessing the long-term impact of treatment. 

## 5. Conclusions

The present pilot study reports the overall improvement of vision-targeted QOL scores in PCV patients after 6 months of treatment, with mental health, role difficulties, social functioning, and distance vision activities being some of the most improved subscales. However, increased sample size and study duration will further enhance our understanding of this improvement. 

## Figures and Tables

**Table 1:** Visual functions of polypoidal choroidal vasculopathy (PCV) study eyes at baseline and 6 months after treatment.

Variable	Baseline	6 Months	f/t	*p*
DVA	0.78 ± 0.31	0.54 ± 0.37	4.756	<0.001
NVA	0.76 ± 0.28	0.46 ± 0.27	5.428	<0.001
CS	0.73 ± 0.26	1.12 ± 0.28	−5.839	<0.001
RS	59.84 ± 23.18	85.42 ± 26.15	−3.929	<0.001

DVA: distance visual acuity; NVA: near visual acuity; CS: contrast sensitivity; RS: reading speed; *p*: *p*-values are statistically significant (*p* < 0.05). f/t: F-test; ±: standard deviation.

**Table 2:** Scores for different subscales of the NEI-VFQ-25 before and after treatment.

Subscale	Baseline QOL	6-Month QOL	*p*-Value
General Health	62.50 ± 18.27	62.16 ± 16.11	0.923
General Vision	52.00 ± 18.64	65.83 ± 20.17	<0.001
Ocular Pain	80.41 ± 16.63	84.16 ± 16.71	0.130
Near Activities	67.77 ± 19.54	70.27±20.37	0.231
Distance Activities	66.66 ± 20.05	71.38 ± 22.17	<0.001
Social Functioning	75.58 ± 19.62	82.50 ± 20.39	0.006
Mental Health	56.45 ± 19.45	67.29 ± 17.18	0.001
Role Difficulties	55.00 ± 22.40	70.83 ± 20.84	0.002
Dependency	63.05 ± 22.39	72.22 ± 20.45	0.004
Driving	31.66 ± 34.56	34.44 ± 38.26	0.217
Color Vision	93.33 ± 13.02	94.16 ± 10.75	0.326
Peripheral Vision	84.16 ± 16.71	85.83 ± 15.65	0.601

Baseline QOL: quality of life at baseline before treatment; 6-Month QOL: Quality of life 6 months after treatment; ±: standard Deviation; *p*: significance value.
